# Feeding Stimulation Ability and Central Effects of Intraperitoneal Treatment of L-Leucine, L-Valine, and L-Proline on Amino Acid Sensing Systems in Rainbow Trout: Implication in Food Intake Control

**DOI:** 10.3389/fphys.2018.01209

**Published:** 2018-08-28

**Authors:** Sara Comesaña, Cristina Velasco, Marta Conde-Sieira, Jesús M. Míguez, José L. Soengas, Sofía Morais

**Affiliations:** ^1^Laboratorio de Fisioloxía Animal, Departamento de Bioloxía Funcional e Ciencias da Saúde, Facultade de Bioloxía and Centro de Investigación Mariña, Universidade de Vigo, Vigo, Spain; ^2^Lucta S.A., Innovation Division, UAB Research Park, Bellaterra, Spain

**Keywords:** rainbow trout, food intake, amino acid sensing, hypothalamus, telencephalon

## Abstract

To continue gathering knowledge on the central regulation of food intake in response to amino acids in teleost fish, using as a model rainbow trout (*Oncorhynchus mykiss*), we evaluated in a first experiment the feeding attractiveness of L-leucine, L-valine, and L-proline offered as an agar gel matrix. In a second experiment, we assessed the effect of intraperitoneal (IP) treatment with the same amino acids on food intake. In a third experiment, we carried out a similar IP administration of amino acids to evaluate the response of amino acid sensing mechanisms in the hypothalamus and telencephalon. Results are discussed in conjunction with an earlier study where leucine and valine were administered intracerebroventricularly (ICV). The attractiveness of amino acids does not appear to relate to their effects on food intake, at least when administrated by-passing ingestion and luminal absorption, since two attractive amino acids resulted in an anorexigenic (Leu) or no effects (Pro) on food intake while a non-attractive amino acid (Val) induced anorexigenic (IP treatment) or orexigenic (ICV treatment) responses. The effects of Leu on food intake might relate to the expression of hypothalamic neuropeptides and result from the direct activation of amino acid sensing systems. In contrast, while valine had few effects on hypothalamic amino acid sensing systems after ICV treatment, a significant amount of parameters become affected by IP treatment suggesting that the effect of Val after IP treatment is indirect. Proline had no relevant effects on amino acid sensing systems, neuropeptide expression, and food intake, which suggest that this amino acid might not have a relevant role in the homeostatic regulation of food intake through hypothalamic mechanisms. In telencephalon, the same amino acid sensing systems operating in hypothalamus appear to be present and respond to Leu and Val, but it is still unclear how they might relate to the control of food intake.

## Introduction

The detection of changes in nutrient levels in vertebrate brain is a fundamental process involved in the homeostatic regulation of food intake (Delgado et al., 2017; Soengas et al., 2018). Homeostatic processes primarily occur in the hypothalamus, which senses metabolic and hormonal signals that inform the brain on the status of energy stores in the periphery, regulating energy intake. Accordingly, several mechanisms of nutrient sensing are present in hypothalamus to detect changes in the levels of glucose, fatty acids, and amino acids (Efeyan et al., 2015; Soengas et al., 2018). The hypothalamus contains two different neuronal populations involved in the integration of metabolic and endocrine signals related to the homeostatic control of food intake: one co-expressing appetite stimulators (orexigenic) neuropeptide Y (NPY) and Agouti-related peptide (AgRP), and another population co-expressing appetite supressors (anorexigenic) pro-opiomelanocortin (POMC) and cocaine- and amphetamine-regulated transcript (CART) (Delgado et al., 2017; Soengas et al., 2018). Thus, an increase in circulating nutrient levels results in decreased NPY/AgRP mRNA abundance, increased mRNA abundance of POMC/CART, and decreased food intake, whereas a fall in nutrient levels leads to opposite changes (Conde-Sieira and Soengas, 2017). The mechanisms linking nutrient sensors with the expression of these neuropeptides are mostly unknown in mammals (Diéguez et al., 2011) and fish (Delgado et al., 2017), although they are believed to include forkhead boxO1 (FoxO1), brain homeobox transcription factor (BSX), and phosphorylated cAMP response element binding protein (CREB).

On the other hand, the hedonic or reward-based regulation of food intake is driven by sensory perception and pleasure, resulting in consumption of highly palatable foods independently of the energy balance status, and can override the homeostatic pathway, inducing animals to overconsume food (Berthoud, 2006; Lutter and Nestler, 2009). Both homeostatic and hedonic systems are linked and activated during feeding although the degree of activation depends on the type of food and physiological status of the animal (Rossi and Stuber, 2017). In contrast to the homeostatic pathway, the mechanisms involved in the regulation of food intake by the hedonic system are scarcely known (Lutter and Nestler, 2009). The brain area in which these mechanisms are likely located in fish is the telencephalon but there are very few available studies concerning this topic (Mueller and Wullimann, 2009; O’Connell and Hofmann, 2011).

Regarding the metabolic regulation of food intake induced by changes in circulating amino acids levels in mammals, it is thought that branched-chain amino acids (BCAA) specifically signal protein availability (Efeyan et al., 2015; Heeley and Blouet, 2016). However, leucine is the unique BCAA whose increased levels are detected by mammalian central amino acid sensing mechanisms and consequently modulate NPY/AgRP and POMC/CART expression to reduce food intake (Heeley and Blouet, 2016; Soengas et al., 2018). This process occurs through not completely understood mechanisms based on (1) activation of BCAA metabolism (Morrison and Laeger, 2015), (2) activation of glutamine metabolism (Jewell and Guan, 2013), (3) activation of mechanistic target of rapamycin (mTOR) (Cavanaugh et al., 2015; Hu et al., 2016) and/or inhibition of AMP-activated protein kinase (AMPK) signaling (Fromentin et al., 2012), and (4) umami taste receptor signaling (Wauson et al., 2012). Taste receptors of the type 1 (T1R) family, beyond their sensory function in oral tissues, are also expressed in brain, where they play an important role as nutrient sensors (Herrera Moro Chao et al., 2016; Lee and Owyang, 2017). Furthermore, there is another mechanism which is activated in cases of deficiency of essential amino acids and elicits an increase in food intake mediated by general control non-derepressible 2 (GCN2) kinase which subsequently phosphorylates eukaryotic initiation factor 2α (eIF2α), leading to reduced global protein synthesis and increased expression of specific genes. One of these genes is system A amino acid transporter 2 (SNAT2), which has been considered to detect changes in BCAA alone, i.e., without previous changes in GCN2 (Hundal and Taylor, 2009). Another gene is sestrin 2 (SESN2), which has been postulated to inhibit mTOR in the absence of leucine (Wolfson and Sabatini, 2017).

In a previous study (Comesaña et al., 2018) on rainbow trout (*Oncorhynchus mykiss*) as a model teleost fish, we demonstrated that intracerebroventricular (ICV) treatment with leucine decreased food intake as expected whereas valine, contrary to mammals, had a clear orexigenic effect. We related the effects on food intake to the presence of amino acid sensing systems (dependent on metabolism of BCAA, metabolism of glutamine, mTOR, T1R receptors, and GCN2) sensitive to leucine in hypothalamus and telencephalon. As for valine, responses of amino acid sensing systems were partially observed in telencephalon but not in hypothalamus. This earlier study led us to hypothesize that valine might be attractive to trout and that stimulation of food intake might have occurred through reward and hedonic mechanisms operating in telencephalon.

Therefore, the first objective of this follow-up study was to confirm the hypothesized hedonic value of valine by performing a feeding attractiveness experiment with amino acids offered as an agar gel matrix, in order to relate the central regulation of food intake by amino acids to their possible feeding stimulation effects. In a second experiment, we assessed the effect of intraperitoneal (IP) treatment with leucine and valine on food intake. These amino acids were initially selected considering that, in mammals, leucine is the unique activator of amino acid sensing systems in brain whereas valine acts as a negative control (Heeley and Blouet, 2016). However, the previous realization of important differences between trout and mammals (Comesaña et al., 2018) led us to consider extending the investigation to a non-BCAA in this study. Proline was was selected for several reasons: it typically has a high palatability in carnivore fish species (Li et al., 2009; Morais, 2017), including rainbow trout (Jones, 1989), it has very diverse and important roles in cell metabolism and physiology, and is considered a conditionally essential amino acid in fish given that rates of endogenous synthesis are inadequate during early life stages, and possibly also in adults (Li et al., 2009; Wu et al., 2011). Furthermore, the requirement of proline for whole-body protein synthesis is the highest of all amino acids, on a per-gram basis (Wu et al., 2011). Finally, in order to establish whether the action of dietary leucine and valine on central amino acid sensors is possibly direct, we carried out a third experiment with a similar IP administration of amino acids to evaluate the response of amino acid sensing mechanisms in the hypothalamus and telencephalon, and compare it with the previous results after ICV administration (Comesaña et al., 2018). In particular, we assessed parameters related to putative amino acid sensing mechanisms based on: (1) metabolism of BCAA, evaluated through branched-chain amino acid aminotransferase (BCAT) activity, and mRNA abundance of branched-chain α-keto acid dehydrogenase E2 subunit (BCKDE2) and branched chain ketoacid dehydrogenase kinase (BCKDK), (2) metabolism of glutamine, evaluated by glutamine synthase (GLS) and glutamate dehydrogenase (GDH) activities, and mRNA abundance of GLS1 and GLS2, (3) mTOR/AMPKα, evaluated by the abundance of their mRNA as well as by their phosphorylation status, (4) T1R receptors, including the umami and sweet taste receptors (which in fish also bind amino acid ligants; Oike et al., 2007), through mRNA abundance of subunits T1R1, T1R2, and T1R3, and (5) GCN2, evaluated by mRNA abundance of eIF2α, SNAT2, and SESN2. Additionally, we also evaluated changes in mRNA abundance of neuropeptides involved in food intake regulation (NPY, AgRP, POMC-A1, and CART), as well as levels and phosphorylation status of proteins (CREB and FoxO1) putatively involved in linking changes in amino acid sensing systems with the expression of neuropeptides. In sum, the experiments reported here will enable to continue gathering knowledge on the central regulation of food intake in response to amino acids in a teleost fish.

## Materials and Methods

### Fish

Rainbow trout were obtained from a local fish farm (A Estrada, Spain) and maintained under laboratory conditions in the Universidade de Vigo for 1 month in 100 L tanks, with 12L:12D photoperiod (lights on at 08:00 h, lights off at 20:00 h), in dechlorinated tap water at 15°C. Fish were fed once daily (09.00 h) to satiety with commercial dry fish pellets (Dibaq-Diproteg SA, Spain) containing 48% crude protein, 14% carbohydrates, 25% crude fat, 11.5% ash, and 20.2 MJ/kg of feed. The experiments described comply with the Guidelines of the European Union Council (2010/63/UE), and of the Spanish Government (RD 55/2013) for the use of animals in research, and were approved by the Ethics Committee of the Universidade de Vigo.

### Experimental Design

#### Feeding Stimulation

Following 1-month acclimation period, fish of 87.6 ± 8.3 g size were randomly assigned to eight 100 L experimental tanks at a density of four fish per tank. In this first experiment, fish were offered pellets of an agar gel matrix containing water alone (control) or with L-leucine, L-valine, or L-proline, as described by Papatryphon and Soares (2000). The test gels were prepared solubilizing agar–agar by heating at a concentration of 2% in 40 mL of deionized water. Before reaching gelatinization, L-leucine, L-valine, or L-proline were added at a final concentration of 0.1 M, and a red dye (4R Ponceau, #199737, Sigma, St. Louis, MO, United States) was added to all gels at a final concentration of 5 μM, to enable visual detection of the pellets. In the control group, the pellets contained only the dye. The gel was poured into Petri dishes and allowed to completely gelatinize. The pellets were cut out to similar size pieces of 3 mm diameter and 3 mm length using a stainless steel tube immediately before feeding. Fish were fed twice daily with the agar pellets in the morning offering fish the same amount of pellets (in excess) in all treatments, when food intake was recorded, and with commercial dry fish pellets (see above) in the afternoon. Agar pellets offered were counted and uneaten pellets were withdrawn and counted to know the number of pellets consumed by all fish in each tank. The test diets were rotated among the tanks every day during 8 days, so that each tank received each tested diet twice. Two replicates of each of the four groups were tested each day, resulting in *n* = 16 tests per treatment. Taste attractiveness of each amino acid was expressed as % of pellets ingested/pellets offered.

#### IP Injection

A second type of experiments was performed using new fish acclimated for at least 1 month and randomly assigned to four 100 L experimental tanks. Fish were fasted for 24 h before treatment to ensure that basal levels of hormones involved in metabolic control were achieved. On the day of the experiment, fish were lightly anesthetized with 2-phenoxyethanol (Sigma, 0.02% v/v) and weighed. Fish received 0.5 mL⋅100 g^-1^ IP injection of saline solution (0.6% NaCl) alone (control) or containing 40 μmol⋅mL^-1^ L-leucine, L-valine, or L-proline. Dose was calculated from the amount of leucine ingested per day by a trout fed a standard commercial diet (Wacyk et al., 2012).

In a first set of experiments, fish of 89.2 ± 3.3 g size were used for the assessment of food intake. This was registered in the whole tank for 3 days before treatment (to evaluate basal level of food intake) and then 6, 24, and 48 h after IP treatment with saline solution alone (control, *n* = 10 fish) or containing L-leucine (*n* = 10 fish), L-valine (*n* = 10 fish), or L-proline (*n* = 10 fish). After feeding, uneaten food and feed waste remaining at the bottom of the conical tanks were withdrawn, dried, and weighed, and this value was used to calculate the amount of food consumed by all fish in each tank, as the difference from the feed offered (de Pedro et al., 1998; Polakof et al., 2008a,b). The experiment was repeated three times and results shown are the mean ± SEM of the three experiments (*N* = 3) each with *n* = 10 fish per treatment in each tank.

In a second set of experiments, fish of 71.8 ± 1.3 g size were IP injected with saline solution alone (control, *n* = 22 fish) or containing L-leucine (*n* = 22 fish), L-valine (*n* = 22 fish), or L-proline (*n* = 22 fish), as described above. After 6 h, fish were lightly anesthetized with 2-phenoxyethanol (Sigma, 0.02% v/v). Blood was collected by caudal puncture with ammonium-heparinized syringes, and plasma samples were obtained after blood centrifugation, deproteinized immediately (using 0.6 M perchloric acid), and neutralized (using 1 M potassium bicarbonate) before freezing on dry ice and storing at -80°C until further assay. Fish were then sacrificed by decapitation, and hypothalamus and telencephalon were dissected, snap-frozen, and stored at -80°C. Ten fish per group were used to measure enzyme activities and metabolite levels, six fish per group were used for the assessment of mRNA levels by qRT-PCR, whereas the remaining six fish per group were used to assess changes in the phosphorylation status of proteins through western blot.

### Assessment of Metabolite Levels and Enzyme Activities

Levels of glucose and lactate in plasma were determined enzymatically using commercial kits (Spinreact, Barcelona, Spain). Total α-amino acids were assessed colorimetrically using the nynhydrin method (Moore, 1968) with alanine as standard.

Samples used to assess tissue metabolite levels were quickly homogenized by ultrasonic disruption in 7.5 vols of ice-cooled 0.6 M perchloric acid, and neutralized with 1 M potassium bicarbonate. The homogenate was centrifuged (10,000 *g*) and the supernatant used to assay tissue metabolites. Tissue total α-amino acids levels were determined colorimetrically as described above for plasma samples.

Samples for enzyme activities were homogenized by ultrasonic disruption with 9 vols of ice-cold buffer consisting of 50 mM Tris (pH 7.6), 5 mM EDTA, 2 mM 1,4-dithiothreitol, and a protease inhibitor cocktail (Sigma). The homogenate was centrifuged and the supernatant used immediately for enzyme assays. Enzyme activities were determined using a microplate reader INFINITE 200 Pro (Tecan, Männedorf, Switzerland). Reaction rates of enzymes were determined by the decrease in absorbance of NADH at 340 nm or, in the case of GLS activity, of Fe-*G*-hydroxy glutamyl complex at 500 nm in acid medium, for which FeCl_3_ 6.7% in HCl 1 N was added after incubation. The reactions were started by the addition of supernatant (10–15 μL) at a pre-established protein concentration, omitting the substrate in control wells (final volume 180–275 μL), and allowing the reactions to proceed at 37°C for pre-established times (3–20 min). Enzyme activities were normalized to protein levels (mg). Protein was assayed in triplicate in homogenates using a microplate reader, according to the bicinchoninic acid method with bovine serum albumin (Sigma) as standard. Enzyme activities were assessed at maximum rates after preliminary tests to determine optimal substrate concentrations. BCAT (*EC* 2.6.1.42), glutamine synthetase (GLS, *EC* 6.3.1.2), and GDH (*EC* 1.4.1.4) activities were determined as previously described (Comesaña et al., 2018).

### Analysis of mRNA Abundance by Real-Time Quantitative PCR

Total RNA of hypothalamus and telencephalon samples was extracted using Trizol reagent (Life Technologies, Grand Island, NY, United States) and subsequently treated with RQ1-DNAse (Promega, Madison, WI, United States); 2 μg total RNA were reverse transcribed using Superscript II reverse transcriptase (Promega) and random hexamers (Promega) in a reaction volume of 20 μL. Gene expression levels were determined by real-time quantitative PCR (RT-qPCR) using the iCycler iQ (BIO-RAD, Hercules, CA, United States). Analyses were performed on 1 μL cDNA (previously diluted 1:1) using MAXIMA SYBR Green qPCR Mastermix (Life Technologies), in a total PCR reaction volume of 15 μL, containing 50–500 nM of each primer. Most transcripts were measured using previously described primers (Comesaña et al., 2018), with the exception of T1R1 and SESN2. For these transcripts, new primers were designed using Primer3 software^1^ from sequences available in GenBank (T1R1, XM_021614421.1; SESN2, XM_021572426.1). A fragment of each sequence containing the amplicon was amplified by conventional PCR and run on a 1.2% agarose gel. The corresponding bands were cut from the gel, purified with the QIAquick Gel Extraction Kit (Qiagen, Hilden, Germany) and sequenced in an Applied Biosystems 3130 (Foster City, CA, United States) in Servicio de Determinación Estructural, Proteómica y Genómica (CACTI-Universidade de Vigo). The obtained sequences satisfactorily matched the reference GenBank sequences. Forward and reverse primers used for each gene expression assay are shown in **Supplementary Table S1**. Thermal cycling was initiated with incubation at 95°C for 90 s using hot-start iTaq DNA polymerase activation followed by 40 cycles, each one consisting of heating at 95°C for 20 s, and specific annealing and extension temperatures (**Supplementary Table S1**) for 20 s. Following the final PCR cycle, melting curves were systematically performed and monitored (55°C temperature gradient at 0.5°C/s from 55 to 94°C) to ensure that only one fragment was amplified. Samples without reverse transcriptase and samples without RNA were run in each qPCR assay as negative controls. Relative expression of the target transcripts was calculated using β-actin and elongation factor 1α (EF1α) as reference genes, which were stably expressed in this experiment, following the Pfaffl (2001) method.

### Western Blot Analysis

Frozen samples were homogenized in 1 mL of buffer containing 150 mM NaCl, 10 mM Tris-HCl, 1 mM EGTA, 1 mM EDTA (pH 7.4), 100 mM sodium fluoride, 4 mM sodium pyrophosphate, 2 mM sodium orthovanadate, 1% Triton X-100, 0.5% NP40-IGEPAL, and 1.02 mg.mL^-1^ protease inhibitor cocktail (Sigma). Tubes were kept on ice during the whole process to prevent protein denaturation. Homogenates were centrifuged at 1,000 *g* for 15 min at 4°C, and supernatants were again centrifuged at 20,000 *g* for 30 min. The resulting supernatants were recovered and stored at -80°C. The concentration of protein in each sample was determined using the Bradford assay with bovine serum albumin as standard. Protein lysates (20 μg) were used for western blotting using appropriate antibodies from (1) Cell Signaling Technology (Leiden, Netherlands): anti-phospho AMPKα (Thr172) ref. #2531, anti-AMPKα ref. #2532, anti-CREB (48H2) ref. #9197, anti-phospho-CREB (Ser133) ref. #9198, anti-phospho-FoxO1 (Thr24) #ref. 9464, anti-FoxO1 (L27) ref. #9454, anti-phospho-mTOR (Ser2448) ref. #5536, and anti-β-tubulin ref. #2146, or (2) Sigma: anti m-TOR ref. #T2949. All these antibodies cross-react successfully with the proteins of interest in rainbow trout (Skiba-Cassy et al., 2009; Sánchez-Gurmaches et al., 2010; Kamalam et al., 2012; Librán-Pérez et al., 2015; Velasco et al., 2016). After washing, membranes were incubated with an IgG-HRP secondary antibody ref. #2015718 (Abcam, Cambridge, United Kingdom) and bands were quantified by Image Lab software version 5.2.1 (BIO-RAD) in a Chemidoc Touch Imaging system (BIO-RAD).

### Statistics

Comparisons among groups were carried out with one-way ANOVA followed by a Student–Newman–Keuls test using the statistical package SigmaStat. Differences were considered statistically significant at *P* < 0.05.

## Results

**FIGURE 1:**
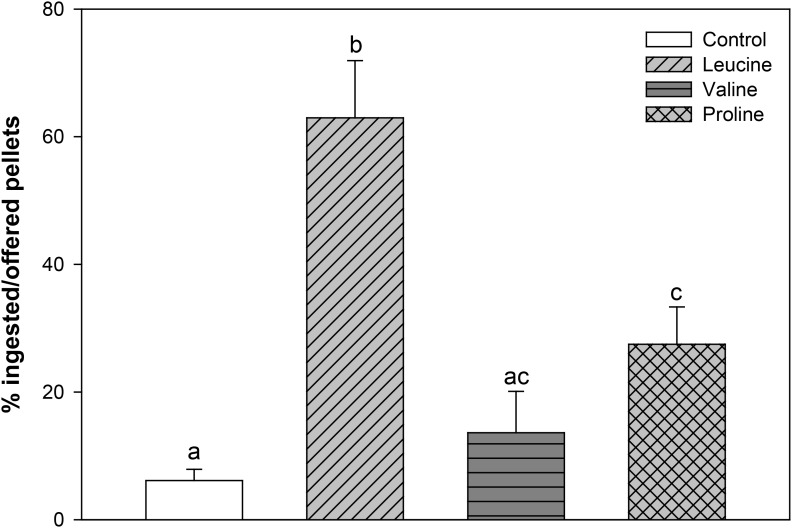
Percentage of ingested pellets, in relation to offered pellets, of rainbow trout fed agar pellets containing water alone (control) or 0.1 M L-leucine, L-valine, or L-proline. The results are shown as mean + SEM of 16 different experiments in which four fish were used per group in each tank. Different letters indicate significant differences (*P* < 0.05) between different groups. *P*-values are: *C* vs *L* < 0.001, *C* vs *V* = 0.511, *C* vs *P* = 0.012, *L* vs *V* < 0.001, *L* vs *P* = 0.005, *V* vs *P* = 0.142.

When fish were offered agar pellets containing 0.1 M of either L-leucine, L-valine, and L-proline, a significantly higher ingestion was obtained with L-leucine and L-proline, which was particularly remarkable for L-leucine, compared with the control group, while L-valine was ingested at a similar level as the control (**Figure 1**).

**FIGURE 2:**
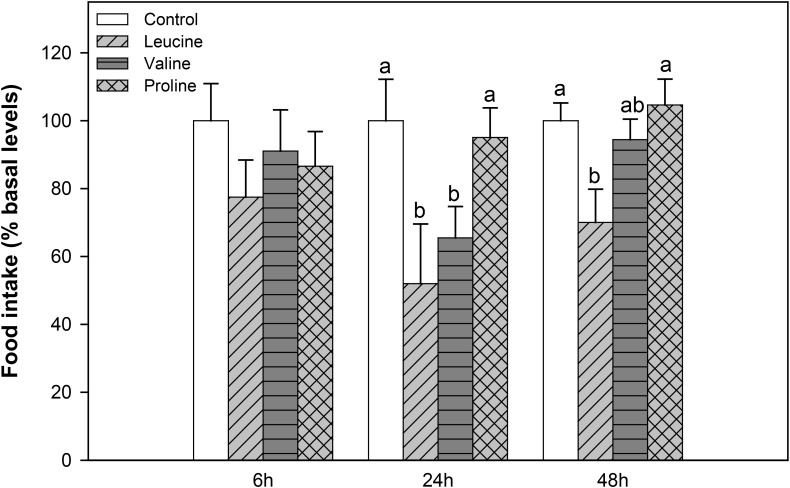
Food intake in rainbow trout 6, 24, and 48 h after intraperitoneal administration of 0.5 mL⋅100 g^-1^ body mass of saline solution alone (control) or containing 40 μmol⋅mL^-1^ of *L*-leucine, 40 μmol⋅mL^-1^ of L-valine, or 40 μmol⋅mL^-1^ of L-proline. Food intake is displayed as mean + SEM of the percentage of food ingested with respect to baseline levels (calculated as the average of food intake in the 3 days previous to the experiment), from three different experiments in which 10 fish were used per group in each tank. Different letters indicate significant differences (*P* < 0.05) between different groups. *P*-values are: at 6 h, = 0.389; at 24 h, *C* vs *L* = 0.017, *C* vs *V* = 0.030, *C* vs *P* = 0.838, *L* vs *V* = 0.412, *L* vs *P* = 0.015, *V* vs *P* = 0.029; and at 48 h, *C* vs *L* = 0.037, *C* vs *V* = 0.817, *C* vs *P* = 0.869, *L* vs *V* = 0.237, *L* vs *P* = 0.039, *V* vs *P* = 0.639.

In the second experiment, food intake decreased significantly 24 h after IP administration with leucine and valine, and remained significantly lower 48 h after treatment with valine, compared to the control and proline treatment. IP injection with proline, on the other hand, did not affect subsequent food intake (**Figure 2**).

**FIGURE 3:**
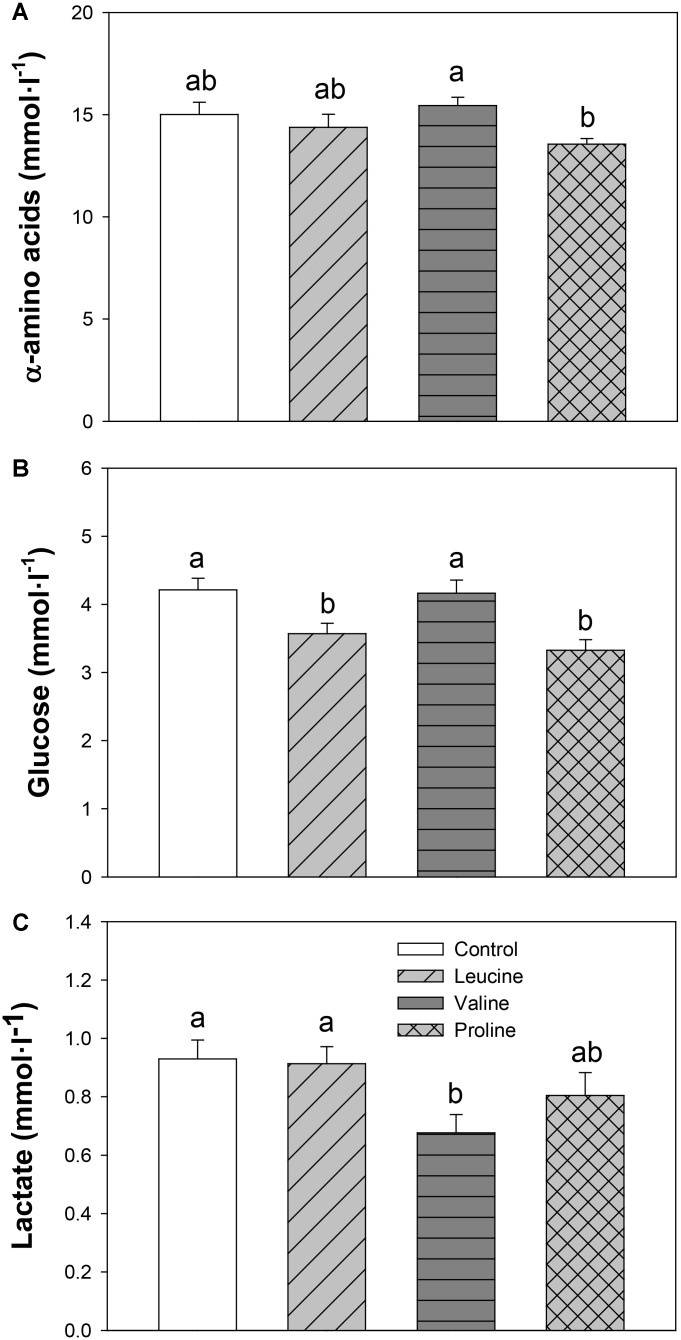
Plasma levels of α-amino acids **(A)**, glucose **(B)**, and lactate **(C)** in rainbow trout 6 h after intraperitoneal administration of 0.5 mL⋅100 g^-1^ body mass of saline solution alone (control) or containing 40 μmol⋅mL^-1^ of L-leucine, 40 μmol⋅mL^-1^ of L-valine, or 40 μmol⋅mL^-1^ of L-proline. Each value is the mean + SEM of *n* = 22 fish per treatment. Different letters indicate significant differences (*P* < 0.05) between different groups. *P*-values are: for α-amino acids **(A)**, *C* vs *L* = 0.364, *C* vs *V* = 0.524, *C* vs *P* = 0.096, *L* vs *V* = 0.267, *L* vs *P* = 0.240, *V* vs *P* = 0.035; for glucose **(B)**, *C* vs *L* = 0.019, *C* vs *V* = 0.832, *C* vs *P* = 0.002, *L* vs *V* = 0.013, *L* vs *P* = 0.305, *V* vs *P* = 0.002; and for lactate **(C)**, *C* vs *L* = 0.856, *C* vs *V* = 0.030, *C* vs *P* = 0.364, *L* vs *V* = 0.027, *L* vs *P* = 0.240, *V* vs *P* = 0.164.

Total α-amino acids in plasma (**Figure 3A**) were not significantly different compared with the control, but the proline group showed decreased values compared with the valine-treated group. Both leucine and proline treatment led to a significant decrease in plasma glucose (**Figure 3B**), compared to the control and valine treatment, and no changes in lactate level compared to the control. Valine had an opposite effect, significantly reducing lactate level in plasma compared to the control, leucine, and proline (only numerically in this case) group (**Figure 3C**).

**FIGURE 4:**
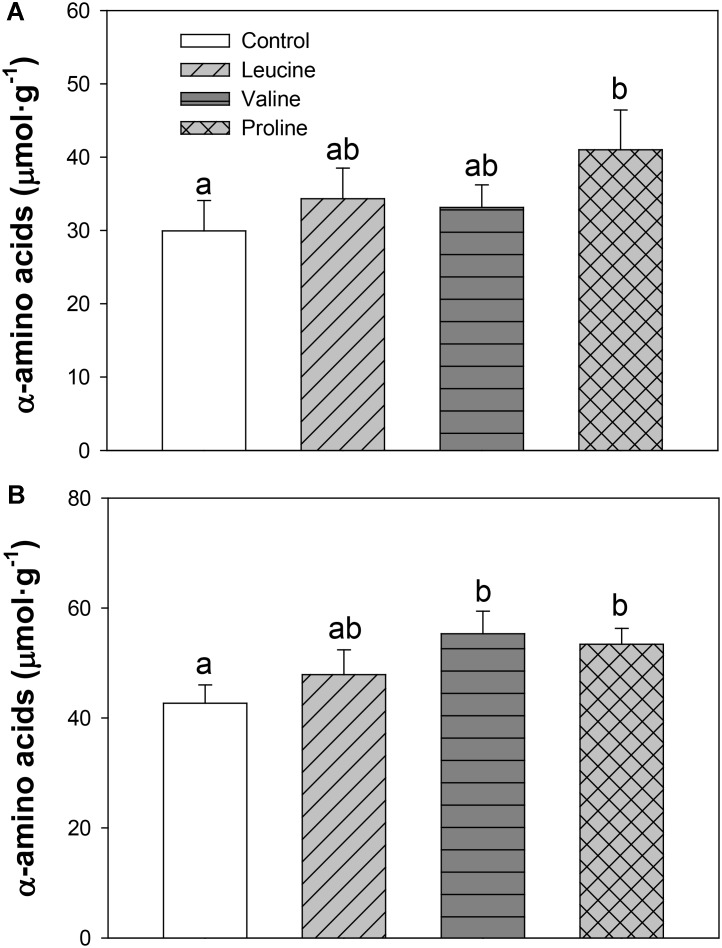
Levels of α-amino acids in hypothalamus **(A)** and telencephalon **(B)** of rainbow trout 6 h after intraperitoneal administration of 0.5 mL⋅100 g^-1^ body mass of saline solution alone (control) or containing 40 μmol⋅mL^-1^ of L-leucine, 40 μmol⋅mL^-1^ of L-valine, or 40 μmol⋅mL^-1^ of L-proline. Each value is the mean + SEM of *n* = 10 fish per treatment. Different letters indicate significant differences (*P* < 0.05) between different groups. *P*-values are: for hypothalamus **(A)**, *C* vs *L* = 0.448, *C* vs *V* = 0.514, *C* vs *P* = 0.037, *L* vs *V* = 0.809, *L* vs *P* = 0.149, *V* vs *P* = 0.287; and for telencephalon **(B)**, *C* vs *L* = 0.338, *C* vs *V* = 0.024, *C* vs *P* = 0.020, *L* vs *V* = 0.212, *L* vs *P* = 0.286, *V* vs *P* = 0.698.

The levels of α-amino acids increased in hypothalamus (**Figure 4A**) with proline treatment compared with the control, while in telencephalon (**Figure 4B**), both valine and proline treatments increased the levels of α-amino acids compared with the control group.

**FIGURE 5:**
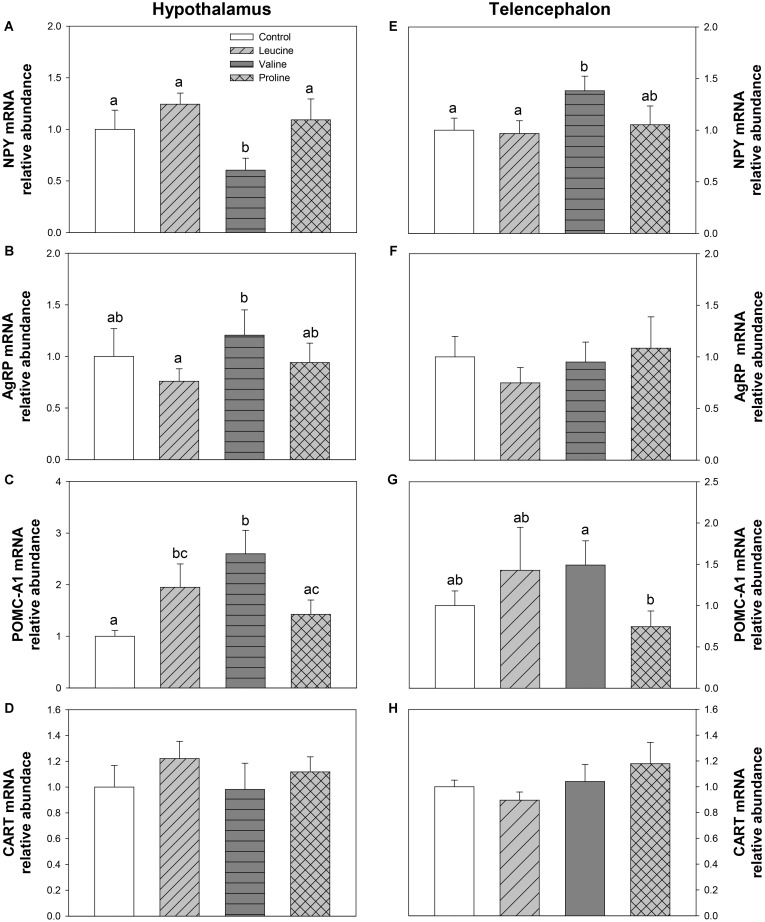
Neuropeptide expression. mRNA levels of NPY **(A,E)**, AgRP **(B,F)**, POMC-A1 **(C,G)**, and CART **(D,H)** in hypothalamus **(A–D)** and telencephalon **(E–H)** of rainbow trout 6 h after intraperitoneal administration of 0.5 mL⋅100 g^-1^ body mass of saline solution alone (control) or containing 40 μmol⋅mL^-1^ of L-leucine, 40 μmol⋅mL^-1^ of L-valine, or 40 μmol⋅mL^-1^ of L-proline. Each value is the mean + SEM of *n* = 6 fish per treatment. Gene expression results are referred to control group and are normalized by EF1α and β-actin expression. Different letters indicate significant differences (*P* < 0.05) between different groups. *P*-values are: for **(A)**, *C* vs *L* = 0.328, *C* vs *V* = 0.019, *C* vs *P* = 0.310, *L* vs *V* = 0.005, *L* vs *P* = 0.561, *V* vs *P* = 0.029; for **(B)**, *C* vs *L* = 0.436, *C* vs *V* = 0.610, *C* vs *P* = 0.868, *L* vs *V* = 0.036, *L* vs *P* = 0.420, *V* vs *P* = 0.410; for **(C)**, *C* vs *L* = 0.041, *C* vs *V* = 0.014, *C* vs *P* = 0.322, *L* vs *V* = 0.215, *L* vs *P* = 0.292, *V* vs *P* = 0.047; for **(D)**, = 0.718; for **(E)**, *C* vs *L* = 0.858, *C* vs *V* = 0.031, *C* vs *P* = 0.812, *L* vs *V* = 0.039, *L* vs *P* = 0.739, *V* vs *P* = 0.130; for **(F)**, = 0.734; for **(G)**, *C* vs *L* = 0.387, *C* vs *V* = 0.165, *C* vs *P* = 0.351, *L* vs *V* = 0.919, *L* vs *P* = 0.189, *V* vs *P* = 0.045; and for **(H)**, = 0.505.

The mRNA abundance of neuropeptides is shown in **Figure 5**. In hypothalamus, leucine treatment induced an increase in POMC-A1 expression (**Figure 5C**) compared with the control group, 6 h after IP administration. In the same tissue, valine treatment decreased mRNA levels of NPY (**Figure 5A**) and increased POMC-A1 expression (**Figure 5C**). Treatment with proline did not affect the expression of neuropeptides in hypothalamus. In telencephalon, the valine group showed significantly increased levels of NPY compared with the control and leucine groups (**Figure 5E**), whereas leucine and proline IP administration did not significantly affect mRNA abundance of neuropeptides in this tissue.

**FIGURE 6:**
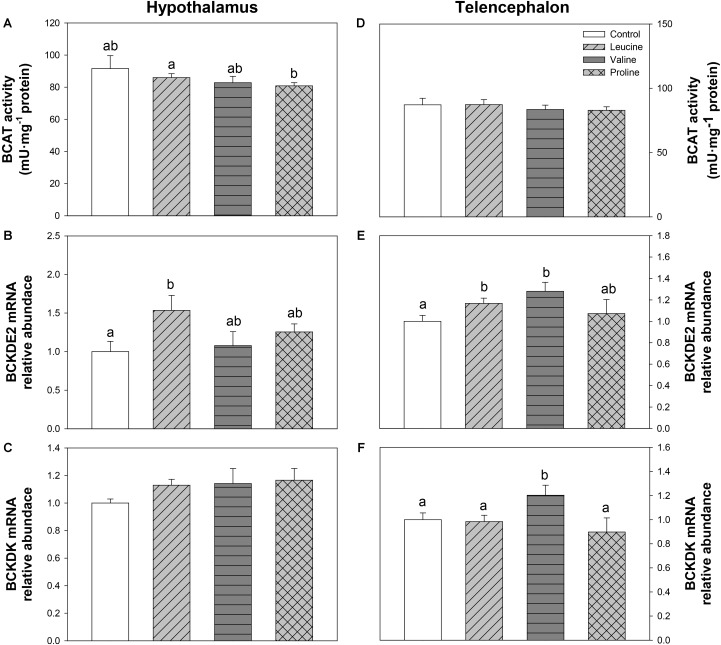
Parameters related to BCAA metabolism. Activity of BCAT **(A,D)**, and mRNA levels of BCKDE2 **(B,E)**, and BCKDK **(C,F)** in hypothalamus **(A–C)** and telencephalon **(D–F)** of rainbow trout 6 h after intraperitoneal administration of 0.5 mL⋅100 g^-1^ body mass of saline solution alone (control) or containing 40 μmol⋅mL^-1^ of L-leucine, 40 μmol⋅mL^-1^ of L-valine, or 40 μmol⋅mL^-1^ of L-proline. Each value is the mean + SEM of *n* = 10 (enzymatic activity) or *n* = 6 (mRNA levels) fish per treatment. Gene expression results are referred to control group and are normalized by EF1α and β-actin expression. Different letters indicate significant differences (*P* < 0.05) between different groups. *P*-values are: for **(A)**, *C* vs *L* = 0.415, *C* vs *V* = 0.255, *C* vs *P* = 0.432, *L* vs *V* = 0.488, *L* vs *P* = 0.033, *V* vs *P* = 0.661; for **(B)**, *C* vs *L* = 0.029, *C* vs *V* = 0.750, *C* vs *P* = 0.167, *L* vs *V* = 0.078, *L* vs *P* = 0.166, *V* vs *P* = 0.442; for **(C)**, = 0.416; for **(D)**, = 0.718; for **(E)**, *C* vs *L* = 0.045, *C* vs *V* = 0.026, *C* vs *P* = 0.626, *L* vs *V* = 0.269, *L* vs *P* = 0.792, *V* vs *P* = 0.199; and for **(F)**, *C* vs *L* = 0.839, *C* vs *V* = 0.028, *C* vs *P* = 0.451, *L* vs *V* = 0.036, *L* vs *P* = 0.489, *V* vs *P* = 0.026.

The parameters assessed related to BCAA metabolism are shown in **Figure 6**. The activity of BCAT in hypothalamus was lower in the group treated with proline than in the leucine treatment (**Figure 6A**). Leucine treatment induced a significant increase in hypothalamic mRNA abundance of BCKDE2 in relation to the control group (**Figure 6B**). In telencephalon, relative to the control, leucine treatment increased the expression of BCKDE2 (**Figure 6E**), whereas valine treatment increased mRNA abundance of both BCKDE2 (**Figure 6E**) and BCKDK (**Figure 6F**).

**FIGURE 7:**
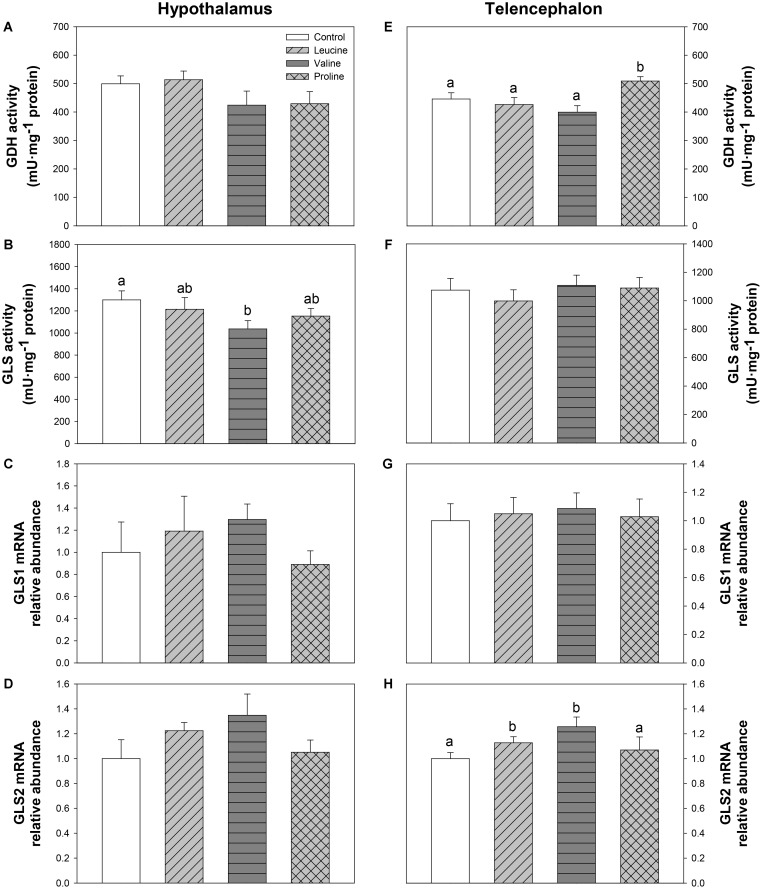
Parameters related to glutamine metabolism. Activities of GDH **(A,E)** and GLS **(B,F)**, and mRNA levels of GLS1 **(C,G)**, and GLS2 **(D,H)** in hypothalamus **(A–D)** and telencephalon **(E–H)** of rainbow trout 6 h after intraperitoneal administration of 0.5 mL⋅100 g^-1^ body mass of saline solution alone (control) or containing 40 μmol⋅mL^-1^ of L-leucine, 40 μmol⋅mL^-1^ of L-valine, or 40 μmol⋅mL^-1^ of L-proline. Each value is the mean + SEM of *n* = 10 (enzymatic activity) or *n* = 6 (mRNA levels) fish per treatment. Gene expression results are referred to control group and are normalized by EF1α and β-actin expression. Different letters indicate significant differences (*P* < 0.05) between different groups. *P*-values are: for **(A)**, = 0.258; for **(B)**, *C* vs *L* = 0.543, *C* vs *V* = 0.026, *C* vs *P* = 0.142, *L* vs *V* = 0.096, *L* vs *P* = 0.631, *V* vs *P* = 0.209; for **(C)**, = 0.640; for **(D)**, = 0.242; for **(E)**, *C* vs *L* = 0.547, *C* vs *V* = 0.298, *C* vs *P* = 0.038, *L* vs *V* = 0.372, *L* vs *P* = 0.032, *V* vs *P* = 0.004; for **(F)**, = 0.764; for **(G)**, = 0.962; and for **(H)**, *C* vs *L* = 0.037, *C* vs *V* = 0.016, *C* vs *P* = 0.385, *L* vs *V* = 0.088, *L* vs *P* = 0.040, *V* vs *P* = 0.102.

**Figure 7** depicts parameters related to glutamate and glutamine metabolism. In the hypothalamus, GLS activity decreased in the valine-treated group compared with the control group (**Figure 7B**). In telencephalon, GDH activity was significantly higher 6 h after IP treatment with proline compared with the other groups (**Figure 7E**), while IP treatment with leucine and valine significantly increased mRNA abundance of GLS2 (**Figure 7H**).

**FIGURE 8:**
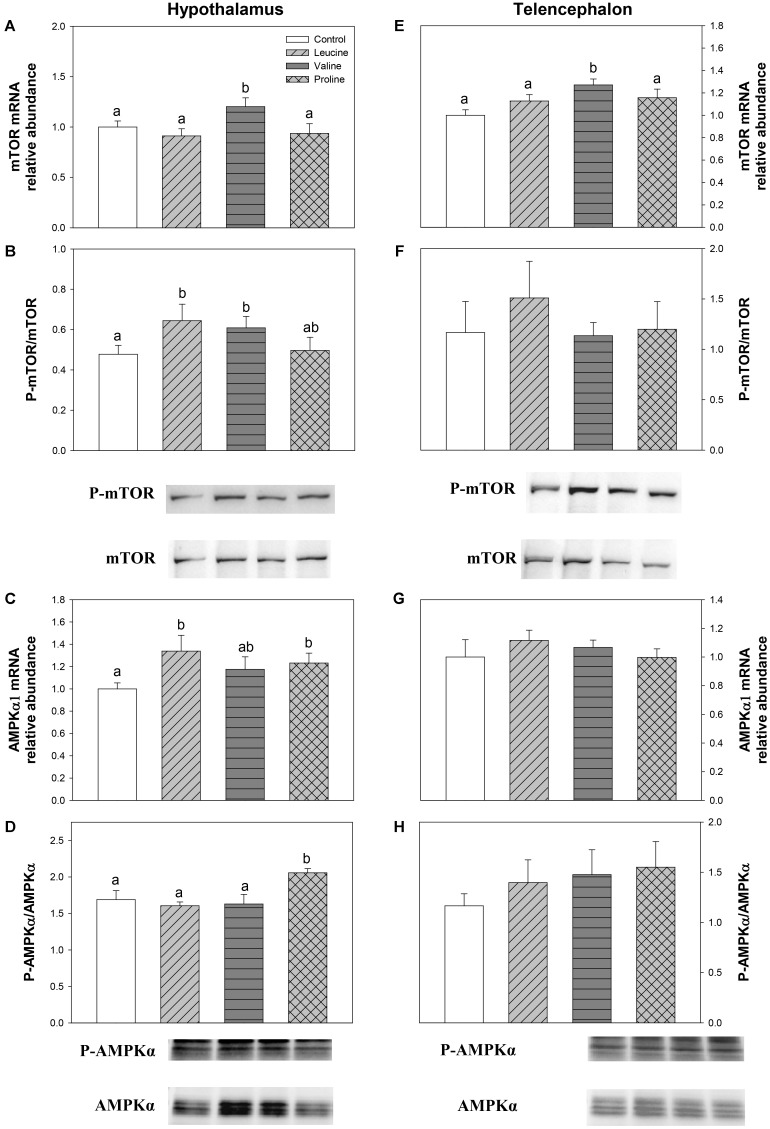
Parameters related to mTOR/AMPKα. mRNA levels **(A,E)** and western blot analysis of phosphorylation status **(B,F)** of mTOR, and mRNA levels **(C,G)** and western blot analysis of phosphorylation status **(D,H)** of AMPKα in hypothalamus **(A–D)** and telencephalon **(E–H)** of rainbow trout 6 h after intraperitoneal administration of 0.5 mL⋅100 g^-1^ body mass of saline solution alone (control) or containing 40 μmol⋅mL^-1^ of L-leucine, 40 μmol⋅mL^-1^ of L-valine, or 40 μmol⋅mL^-1^ of L-proline. Each value is the mean + SEM of *n* = 6 fish per treatment. Gene expression results are referred to control group and are normalized by EF1α and β-actin expression. In western blot analysis, 20 μg of total protein were loaded on the gel per lane. Western blots were performed on six individual samples per treatment, and a representative blot per treatment is shown here. Western blot graphs **(B,F,D,H)** represent the ratio between the phosphorylated protein and the total amount of the target protein. Different letters indicate significant differences (*P* < 0.05) between different groups. *P*-values are: for **(A)**, *C* vs *L* = 0.375, *C* vs *V* = 0.033, *C* vs *P* = 0.573, *L* vs *V* = 0.023, *L* vs *P* = 0.854, *V* vs *P* = 0.036; for **(B)**, *C* vs *L* = 0.037, *C* vs *V* = 0.015, *C* vs *P* = 0.821, *L* vs *V* = 0.729, *L* vs *P* = 0.091, *V* vs *P* = 0.126; for **(C)**, *C* vs *L* = 0.032, *C* vs *V* = 0.165, *C* vs *P* = 0.036, *L* vs *V* = 0.389, *L* vs *P* = 0.561, *V* vs *P* = 0.711; for **(D)**, *C* vs *L* = 0.818, *C* vs *V* = 0.822, *C* vs *P* = 0.033, *L* vs *V* = 0.537, *L* vs *P* < 0.001, *V* vs *P* = 0.032; for **(E)**, *C* vs *L* = 0.164, *C* vs *V* = 0.029, *C* vs *P* = 0.102, *L* vs *V* = 0.039, *L* vs *P* = 0.731, *V* vs *P* = 0.045; for **(F)**, = 0.786; for **(G)** = 0.681; and for **(H)** = 0.638.

The expression and phosphorylation status of mTOR and AMPKα are displayed in **Figure 8**. In hypothalamus, leucine treatment was responsible for a significant increase in mTOR phosphorylation status (**Figure 8B**) and AMPKα1 mRNA abundance (**Figure 8C**) compared with the control. Valine treatment, on the other hand, increased both mRNA abundance (**Figure 8A**) and phosphorylation status (**Figure 8B**) of mTOR, while proline treatment increased mRNA abundance (**Figure 8C**) and phosphorylation status (**Figure 8D**) of AMPKα. In telencephalon, the only significant effect was observed in mTOR expression, which was higher in valine IP-treated fish than in the other groups (**Figure 8E**).

**FIGURE 9:**
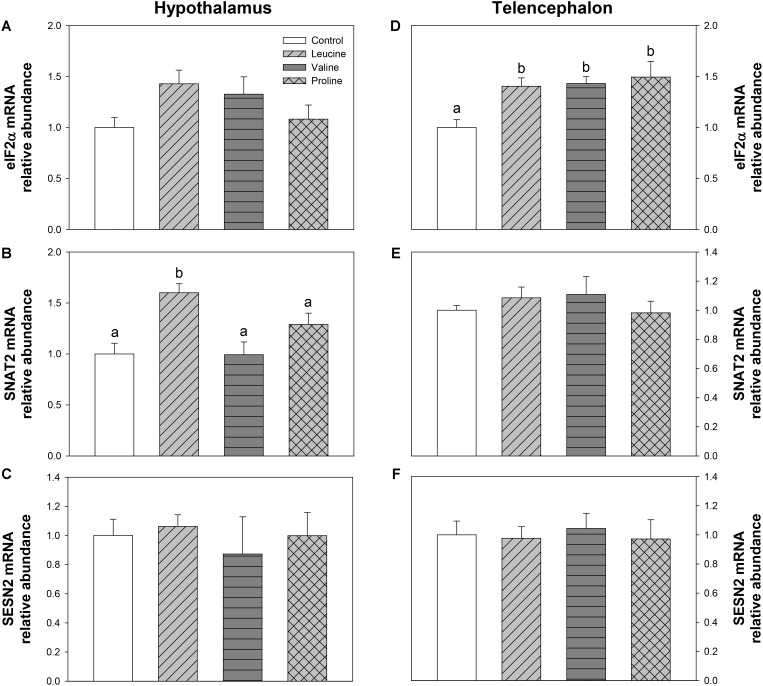
Parameters mediated by GCN2. mRNA levels of eIF2α **(A,D)**, SNAT2 **(B,E)**, and SESN2 **(C,F)** in hypothalamus **(A–C)** and telencephalon **(D–F)** of rainbow trout 6 h after intraperitoneal administration of 0.5 mL⋅100 g^-1^ body mass of saline solution alone (control) or containing 40 μmol⋅mL^-1^ of L-leucine, 40 μmol⋅mL^-1^ of L-valine, or 40 μmol⋅mL^-1^ of L-proline. Each value is the mean + SEM of *n* = 6 fish per treatment. Gene expression results are referred to control group and are normalized by EF1α and β-actin expression. Different letters indicate significant differences (*P* < 0.05) between different groups. *P*-values are: for **(A)**, = 0.104; for **(B)**, *C* vs *L* = 0.002, *C* vs *V* = 0.962, *C* vs *P* = 0.101, *L* vs *V* = 0.008, *L* vs *P* = 0.043, *V* vs *P* = 0.251; for **(C)**, = 0.882; for **(D)**, *C* vs *L* = 0.014, *C* vs *V* = 0.025, *C* vs *P* = 0.018, *L* vs *V* = 0.850, *L* vs *P* = 0.803, *V* vs *P* = 0.662; for **(E)**, = 0.691; and for **(F)**, = 0.958.

The parameters related to mechanisms mediated by GCN2 are shown in **Figure 9**. In hypothalamus, SNAT2 mRNA abundance was significantly increased in the leucine-treated group compared with the remaining treatments (**Figure 9B**). In telencephalon, eIF2α expression (**Figure 9D**) was significantly higher in leucine, valine, and proline-treated groups than in the control.

**FIGURE 10:**
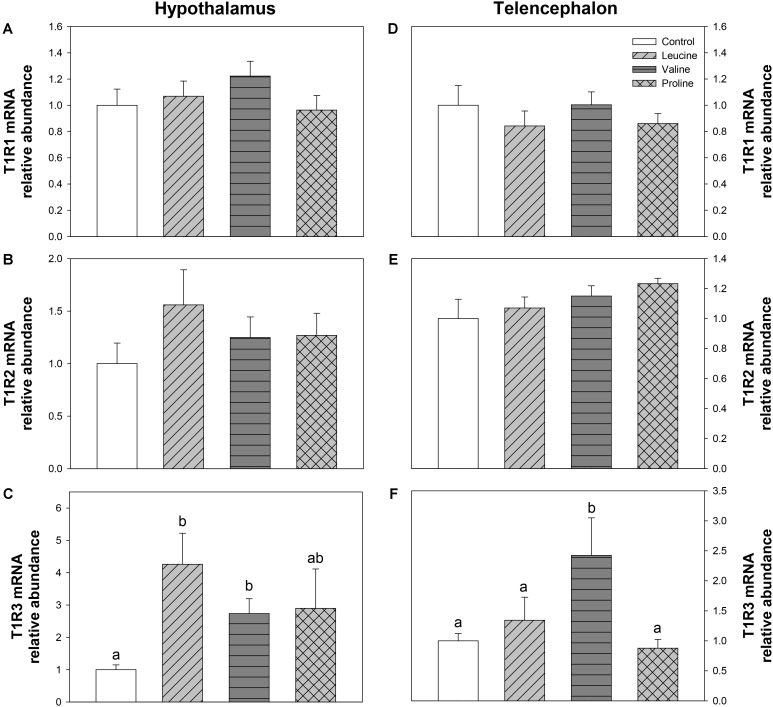
Expression of T1R family taste receptors. mRNA levels of T1R1 **(A,D)**, T1R2 **(B,E)**, and T1R3 **(C,F)** in hypothalamus **(A–C)** and telencephalon **(D–F)** of rainbow trout 6 h after intraperitoneal administration of 0.5 mL⋅100 g^-1^ body mass of saline solution alone (control) or containing 40 μmol⋅mL^-1^ of L-leucine, 40 μmol⋅mL^-1^ of L-valine, or 40 μmol⋅mL^-1^ of L-proline. Each value is the mean + SEM of *n* = 6 fish per treatment. Gene expression results are referred to control group and are normalized by EF1α and β-actin expression. Different letters indicate significant differences (*P* < 0.05) between different groups. *P*-values are: for **(A)**, = 0.403; for **(B)**, = 0.463; for **(C)**, *C* vs *L* = 0.015, *C* vs *V* = 0.029, *C* vs *P* = 0.386, *L* vs *V* = 0.202, *L* vs *P* = 0.414, *V* vs *P* = 1; for **(D)**, = 0.642; for **(E)**, = 0.346; and for **(F)**, *C* vs *L* = 0.508, *C* vs *V* = 0.037, *C* vs *P* = 0.813, *L* vs *V* = 0.043, *L* vs *P* = 0.571, *V* vs *P* = 0.035.

Expression of T1R taste receptors was also measured, and is shown in **Figure 10**. Only the expression of T1R3 was significantly affected, in both brain regions: in hypothalamus, mRNA abundance of T1R3 was increased by leucine and valine treatment (**Figure 10C**) compared with the control group, while in telencephalon, only valine treatment enhanced T1R3 expression, above the remaining groups (**Figure 10F**).

**FIGURE 11:**
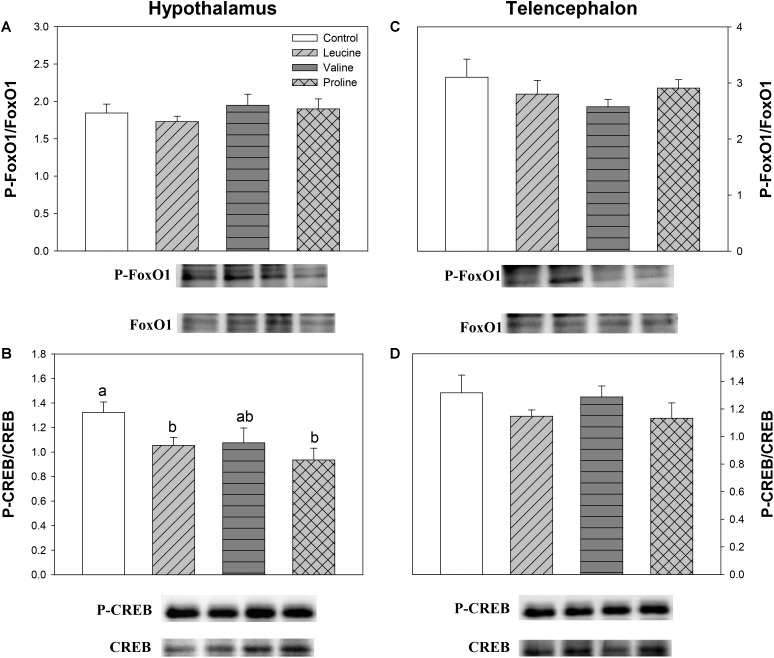
Parameters related to the modulation of transcription factors. Western blot analysis of phosphorylation status of FoxO1 **(A,C)** and CREB **(B,D)** in the hypothalamus **(A,B)** and telencephalon **(C,D)** of rainbow trout 6 h after intraperitoneal administration of 0.5 mL⋅100 g^-1^ body mass of saline solution alone (control) or containing 40 μmol⋅mL^-1^ of L-leucine, 40 μmol⋅mL^-1^ of L-valine, or 40 μmol⋅mL^-1^ of L-proline. Twenty micrograms of total protein were loaded on the gel per lane. Western blots were performed on six individual samples per treatment, and a representative blot per treatment is shown here. Graphs represent the ratio between the phosphorylated protein and the total amount of the target protein. Each value is the mean + SEM of *n* = 6 fish per treatment. Different letters indicate significant differences (*P* < 0.05) between different groups. *P*-values are: for **(A)**, = 0.655; for **(B)**, C vs *L* = 0.038, C vs *V* = 0.104, C vs *P* = 0.017, *L* vs *V* = 0.888, *L* vs *P* = 0.334, *V* vs *P* = 0.402; for **(C)**, = 0.490; and for **(D)**, = 0.341.

Finally, **Figure 11** displays the phosphorylation status of CREB and FoxO1. Significantly differences were only found in hypothalamus, where the ratio of phoshorylated and unphosphorylated forms of CREB decreased in the groups treated with leucine and proline (and tendentially in the valine group) compared with the control (**Figure 11B**).

## Discussion

### Differential Amino Acid Attractiveness Inducing Differences in Feed Intake

Amino acids are important attractants and feeding effectors in all teleost fishes, being detected both by the sense of smell and taste. Olfaction tends to be a more evolutionarily conserved response, involved in other basic functions besides feeding (e.g., kin-nonkin recognition, prey–predator interactions, and territory or homing recognition), while the gustatory system is considered the main determinant of the feeding behavior, providing the final evaluation of the sensorial and nutritional properties of the food, and determining its final consumption (Goh and Tamura, 1980; Hara, 1994). For this reason, the taste spectra of amino acids eliciting stimulatory or deterrent feeding responses is highly species-specific (Hara, 1994; Morais, 2017). Salmonids, including rainbow trout, belong to a group of teleosts which show a narrow range of taste response, responding only to a few amino acids (Marui et al., 1983; Hara, 1994; Kohbara and Caprio, 2001).

Previously (Comesaña et al., 2018), we described the presence of central amino acid sensing mechanisms in rainbow trout brain involved in the regulation of food intake, sharing some similarities with the mammalian system. However, the ICV treatment with valine had a clear and surprising orexigenic effect that was not related to the activation of amino acid sensing in hypothalamus but might have involved the telencephalon, which has been suggested as a possible center for hedonic or reward-based regulation of food intake in fish (Mueller and Wullimann, 2009; O’Connell and Hofmann, 2011). Contrary to leucine, valine has not been previously reported as stimulating feed intake in rainbow trout (Jones, 1989), but studies evaluating its attractiveness are scarce and variation of gustatory specificity of different strains of rainbow trout to amino acid stimuli has been reported (Hara et al., 1999). Therefore, we decided to evaluate the feeding response to leucine and valine by performing a feeding trial in the present study, which should link to their hedonic value. We have also extended this work to include proline, as it has been reported as the most potent amino acid eliciting a gustatory response in both electrophysiological (Marui et al., 1983; Kohbara and Caprio, 2001) and behavioral (Jones, 1989) studies with rainbow trout.

Our results indicate that 0.1 M of leucine, followed by proline, stimulated the consumption of agar pellets, whereas valine did not affect feeding. These results are consistent with a previous feeding behavioral study, where both L-proline and L-leucine were highly effective gustatory compounds, while there was no response to valine (Jones, 1989). The higher response to leucine than to proline was somewhat surprising but this could be related to differences in sensitivity (i.e., threshold of activation of taste nerves) to these amino acids. For instance, proline was the most effective compound at 10^-4^ M but was not found active at 10^-5^ M (Jones, 1989). Therefore, our previous results with valine (Comesaña et al., 2018) cannot be justified by the activation of a putative reward system in the telencephalon and remain unexplained at present. Clearly, many more studies are necessary on the different brain areas and mechanisms possibly regulating food intake in fish in response to amino acids.

### Effects of Amino Acids on Food Intake and Neuropeptide mRNA Abundance

Important differences were observed in the response of several of the parameters assessed in this study (discussed in this and following sections), not only in relation to the control but also between the different amino acid treatments, which validates that the amino acids administered via IP reached the brain in sufficient concentration.

The IP treatment with leucine resulted in a prolonged decrease in food intake (24 and 48 h post-treatment), while valine reduced food intake 24 h after treatment. Administration of proline via IP, on the other hand, did not affect food intake at the times assessed. No other studies are available in fish regarding the effects of IP treatment with any amino acid on food intake, but if we compare these results with those after ICV treatment (Comesaña et al., 2018), we observe that leucine produces similar inhibitory effects, whereas valine has contrary effects. It would therefore appear that leucine functions as a signal of nutrient or energy availability to reduce food intake, whereas valine might have different roles. It is important to highlight that valine has no effects on food intake when administered ICV in mammals (Cota et al., 2006; Blouet and Schwartz, 2012) and that physiological changes in plasma leucine concentration do not lead to a decrease in food intake in mammals (Jordi et al., 2013), even if brain leucine levels are well established as an anorectic signal (Heeley and Blouet, 2016). Therefore, even if amino acid sensing systems are operational in fish and coupled to neuropeptide expression, as in mammals, their regulation of food intake in response to circulating amino acids is clearly different.

Interestingly, food intake after IP treatment with amino acids was not related to their attractiveness, since both leucine, which was highly efficient in inducing feeding, and valine, which did not affect the feeding response, decreased food intake after IP treatment. Thus, at least for the amino acids investigated in the present study, the homeostatic regulation of food intake appears to be independent from the palatability of these nutrients, although further studies, extending this investigation to other amino acids, would be desirable to establish this.

In order to determine the mechanisms underlying the regulation of food intake, we evaluated the expression of neuropeptides (AgRP, NPY, POMC, and CART) involved in the metabolic control of food intake 6 h after IP treatment with amino acids. In hypothalamus, the anorectic effect of leucine and valine is consistent with the increase in POMC-A1 mRNA abundance produced by both amino acids and the decrease observed in NPY abundance by valine in that area, whereas proline did not induce any significant changes. In general, the response produced by leucine or valine could relate to the homeostatic control of food intake through the expression of neuropeptides in the hypothalamus, in a way similar to that observed after ICV treatment in the same species for leucine but not for valine (Comesaña et al., 2018).

In telencephalon, the increase in mRNA abundance of the orexigenic NPY in trout treated with valine and the downregulation of the anorexigenic POMC-A1after IP administration of proline is not consistent with changes in food intake. The function of these neuropeptides in fish telencephalon is unknown and, based on this and in our previous ICV study (Comesaña et al., 2018), evidence is starting to accumulate that it is probably different from that in hypothalamus (Soengas et al., 2018), and likely also unrelated to the reward system, at least when amino acids are sensed after IP or ICV administration, by-passing ingestion, and luminal absorption.

### Differential Activation of Amino Acid Sensing Systems in Hypothalamus

Multiple amino acid sensors in different areas of the brain, and particularly in the hypothalamus, can signal changes in concentrations of circulating amino acids and modulate feeding in mammals. In order to determine whether similar mechanisms operate in trout hypothalamus, we evaluated the responses of several amino acid sensing systems to a peripheral (post-absorptive) increase in amino acid levels. Furthermore, to establish whether the effects of dietary amino acids are direct, or can be otherwise affected by interactions with other molecules or pathways, hepatic or extra-hepatic metabolism, and/or differential uptake through the blood–brain barrier, we compare results from the present study, after IP administration, with those previously observed after ICV administration of leucine and valine (Comesaña et al., 2018).

The increase in body circulating leucine levels resulted in a higher mRNA abundance of BCKDE2 in this study, which was consistent with previous results after ICV administration (Comesaña et al., 2018), thus supporting the responsiveness of hypothalamic amino acid sensing system dependent on BCAA metabolism. In contrast, IP administration of leucine did not produce any effect on the parameters related to glutamine and glutamate metabolism, while ICV treatment enhanced GLS2 expression (Comesaña et al., 2018). On the other hand, leucine produced an increase in mRNA abundance of T1R3 and a non-significant increase in T1R2, which is in complete agreement with the results we previously observed after ICV treatment (Comesaña et al., 2018). The amino acid sensing system dependent on mTOR also appears to be activated by leucine in rainbow trout hypothalamus since an increased phosphorylation status of this protein was measured after leucine treatment, as observed after ICV treatment (Comesaña et al., 2018). However, we observed an increase in mRNA abundance of AMPKα1 in hypothalamus, which contradicts our previous results where no changes were observed after ICV administration (Comesaña et al., 2018) and mammalian studies, where a rise in leucine levels has been reported to induce a decrease in AMPK levels (Cota et al., 2007) and AMPK activity (Ropelle et al., 2008). Since we evaluated the expression of AMPKα1, there is the possibility that AMPKα2 is the form involved in food intake regulation in fish as suggested in mammals (López, 2017). The mechanism dependent on GCN2 also appears to respond to leucine treatment, based on the increase in mRNA abundance of SNAT2, in a way comparable to that observed after ICV treatment (Comesaña et al., 2018). Finally, we have also assessed changes in mRNA abundance of SESN2 for the first time in fish hypothalamus. Although related to the GCN2 kinase pathway, SESN2 can be considered as a leucine sensor. SESN2 negatively regulates mTOR but leucine specifically inactivates SESN2, hence enabling mTOR signaling (Lee et al., 2017; Wolfson and Sabatini, 2017). No changes were observed in SESN2 mRNA abundance after treatment with any of the tested amino acids. In summary, leucine IP treatment generally induced changes in amino acid sensing systems comparable to those we previously observed after ICV administration (Comesaña et al., 2018). In some cases, however, the effects were of lower magnitude and this might relate to the lower levels of leucine in brain after IP, compared to ICV treatment. The fact that results are comparable between IP and ICV treatments allow us to suggest that the action of leucine on sensing mechanisms is direct and might relate to the homeostatic control of food intake in a way comparable to that demonstrated in mammals (Efeyan et al., 2015; Heeley and Blouet, 2016).

Similarly to leucine, valine treatment induced significant effects in mRNA expression and phosphorylation status of mTOR and expression of T1R3 in hypothalamus after IP treatment, but did not affect parameters related to BCAA or glutamine metabolism, and mechanisms related to GCN2. Therefore, even if the effects are not as marked as with leucine, valine appears to be able to activate at least some amino acid sensing systems in hypothalamus after IP treatment. These results are inconsistent with those we previously observed after ICV treatment (Comesaña et al., 2018), which suggests that the effects of valine treatment in the present study must result from an indirect action of peripheral valine inducing changes in unknown molecules or mechanisms which in turn signal the hypothalamus.

Discrepancies in the central actions of leucine and valine in fish, in terms of being direct or indirect, could relate to differences in their peripheral metabolism. Despite many similarities, associated with the fact that they are both BCAA, a main distinction which could be of relevance to the results is that leucine is a ketogenic amino acid, while valine is a glucogenic amino acid, as is proline (Jürss and Bastrop, 1995). In fact, differential effects in metabolism have been hinted in this study by the opposite effects observed in glucose and lactate levels in plasma, being glucose significantly reduced in the leucine and proline treatments (compared to the control and valine groups) and lactate reduced in the valine treatment (compared to the control and leucine groups). It is noteworthy that, in mammals, hypothalamic sensing of leucine, valine, and proline is known to modulate glucose metabolism, inhibiting endogenous glucose production (Arrieta-Cruz et al., 2013, 2016). Furthermore, increases in circulating levels of both leucine and proline have also been reported to lower circulating glucose levels (Su et al., 2012; Arrieta-Cruz et al., 2013). Although establishing glucoregulatory effects of central amino acid sensing was not the objective of the present study, it is interesting to note that this mechanism might also be conserved in fish for leucine, while valine again shows a different effect than in mammals.

On the other hand, the possibility for having interactions or antagonisms between different BCAA, particularly when dosed at supra-physiological amounts, has been often reported in mammals, including humans, and birds. In particular, high levels of leucine intake have been described to substantially lower circulating levels of valine and isoleucine but, in contrast, intakes of these BCAA have little influence on the concentration of plasma free leucine (Swendseid et al., 1965; D’Mello and Lewis, 1970). This type of interaction has also been examined in fish, although results are not always consistent (Wilson and Halver, 1986). In a classic study with channel catfish, it was suggested that a nutritional interaction exists between BCAA and that leucine affects the tissue uptake and/or catabolism of valine and isoleucine (Robinson et al., 1984). In rainbow trout, antagonism induced by excess leucine in the diet has also been reported, reducing free valine and isoleucine concentrations in plasma, muscle, and liver, with adverse effects on growth and protein utilization (Yamamoto et al., 2004). More interestingly, excess dietary leucine levels clearly reduced feed intake under self-feeding conditions, which ties well with results from this and our previous study (Comesaña et al., 2018) showing an anorectic effect of leucine administered IP and ICV. However, such interactions cannot explain why valine is also capable of eliciting changes in feed intake in trout, unlike mammals (Heeley and Blouet, 2016), with opposite effects depending on whether it is administered peripherally or centrally. Further studies are clearly needed in order to establish the role of valine in the control of food intake, as well as the mechanism(s) through which it exerts such effects.

The IP administration of proline did not affect most of the hypothalamic amino acid sensors that were assessed, except for an increase in mRNA abundance and phosphorylation status of AMPKα, and a decrease in the phosphorylation of CREB. It would therefore seem that either proline or its metabolites are capable of reaching the hypothalamus, as demonstrated also by the significant increase in the concentration of α-amino acids in this compartment 6 h after IP administration, compared to the control, but the effects are likely unrelated to the control of food intake.

### Differential Activation of Amino Acid Sensing Systems in Telencephalon

Treatment with leucine induced changes that in general are compatible with an activation of some amino acid sensing systems in telencephalon. These included parameters related to BCAA metabolism (increased mRNA abundance of BCKDE2), glutamine metabolism (increased mRNA abundance of GLS2) and mediated by GCN2 (increased mRNA abundance of elF2α). It seems therefore that leucine is detected in telencephalon in a comparable way to hypothalamus, although more effects were noted when administered ICV (Comesaña et al., 2018). Valine treatment also induced changes that relate to the activation of a few amino acid sensing systems in telencephalon, such as increased mRNA abundance of BCKDE2, BCKDK, GLS2, mTOR, elF2α, and T1R3. The finding that valine elicited more effects than leucine may relate to the higher increase in amino acid levels reached in telencephalon after treatment with valine compared with leucine. As observed in hypothalamus, proline did not exert major effects in telencephalon, despite the increase in total amino acid levels in the area, except an enhancement of GDH enzymatic activity and increased expression of elF2α.

This study confirms that amino acid sensing systems appear to be operative in telencephalon, providing this area with information about circulating levels of amino acids, with different results depending on the type of amino acid. The lack of correlation between the activation of these sensing systems and the feeding stimulation ability of the tested amino acids suggests that these telencephalic mechanisms might relate to still unknown aspects and not to the hedonic control of food intake, as we initially hypothesized (Comesaña et al., 2018).

### Possible Mechanisms Linking Amino Acid Sensing and Food Intake Regulation

In mammals, the activation of nutrient sensing systems influences the production of neuropeptides involved in food intake control through the modulation of transcription factors such as CREB, FoxO1, and BSX (Diéguez et al., 2011; Delgado et al., 2017). However, information on these pathways is still very scarce, especially concerning amino acids.

In the present study, we observed that leucine and proline induced a decrease in the phosphorylation status of CREB in hypothalamus, and a similar trend was noticeable for valine. A similar decrease has been observed in hypothalamus of rainbow trout after treatment with other nutrients, like glucose (Otero-Rodiño et al., 2017), oleate or octanoate (Velasco et al., 2017). This transcription factor is known to stimulate NPY/AgRP and to inhibit POMC/CART mRNA abundance (Varela et al., 2011) but changes induced by amino acids might relate to other pathways, considering how all the tested amino acids tended to affect this gene in a comparable manner, including proline, which had no effects on amino acid sensing systems, neuropeptide expression, and food intake after IP administration.

FoxO1 displayed no changes after treatment with any amino acid, in agreement with the lack of effects we also observed after ICV treatment (Comesaña et al., 2018). The phosphorylation status of this transcription factor usually increases after treatment with other nutrients like glucose or fatty acids, as also demonstrated in hypothalamus of rainbow trout (Otero-Rodiño et al., 2017; Velasco et al., 2017). It seems, therefore, that at least part of the mechanisms involved in linking amino acid sensing with the regulation of food intake through changes in the expression of neuropeptides are not the same when comparing amino acids and other nutrients, at least in rainbow trout. In the case of valine, however, the lack of effects on transcription factors which are putatively involved in the control of neuropeptide expression is not surprising, considering that valine did not affect neuropeptide mRNA abundance.

In telencephalon, no changes were observed in transcription factors, supporting our hypothesis that this area is not involved in the homeostatic regulation of food intake. From the coordinated mechanisms responsible for this regulation in hypothalamus (amino acid sensors, transcription factors, and neuropeptide expression), only amino acid sensors appeared to show some response in telencephalon.

### Perspectives and Significance

As a whole, the present and our previous (Comesaña et al., 2018) study provide information about a complex picture of differential effects of amino acids modulating food intake through homeostatic and probably non-homeostatic (presently unknown) mechanisms in central areas (hypothalamus and telencephalon) of rainbow trout. Evidence so far in rainbow trout supports the notion that hypothalamic modulation of food intake through amino acid sensing systems responds to BCAA, and specifically to leucine, in fish as in mammals (Heeley and Blouet, 2016). This could be expected considering that in fish, just as in mammals, the highest correlation between dietary and postprandial systemic free amino acids composition is observed for essential amino acids (Jürss and Bastrop, 1995) and BCAA show the highest changes in plasma amino acid pool in periods of fasting as well as after feeding (Navarro et al., 1997). On the contrary, non-essential amino acids are more quickly metabolized and converted to a great extent. Therefore, BCAA make much more biological sense as indicators of nutrient and energy availability. However, even if amino acid sensing systems are operative in fish and coupled to neuropeptide expression, as in mammals, their regulation of food intake in response to circulating amino acids is clearly different. Contrary to mammals which respond only to leucine, food intake in rainbow trout was also affected by treatment with valine (even if the outcome was different depending on whether it was administered via ICV or IP). Moreover, the action of leucine on sensing mechanisms appears to be direct and relate to the homeostatic control of food intake, while the actions of valine appear to result from an indirect action of peripheral valine inducing changes in unknown molecules or mechanisms which in turn signal the hypothalamus. Further studies are clearly needed in order to establish the role of valine in the control of food intake, as well as the mechanism(s) through which it exerts such effects.

On the other hand, this study confirms that amino acid sensing systems appear to be operative in telencephalon, although the lack of correlation between the activation of these sensing systems and the attractiveness or feeding stimulatory ability of the tested amino acids suggests that these telencephalic mechanisms might relate to still unknown aspects and not to the hedonic control of food intake, as we initially hypothesized (Comesaña et al., 2018). Furthermore, at least for the amino acids investigated in the present study, the homeostatic regulation of food intake appears to be independent on the palatability of these nutrients, at least when amino acids are sensed after IP or ICV administration. We cannot discard, however, that highly palatable amino acids exert hedonic effects by activating the reward system through peripheral (pre-absorptive) actions.

## Author Contributions

SC, SM, and JS conceived and designed the research. SC, CV, JM, and MC-S performed the experiments. SC, CV, and MC-S analyzed the data. SC and JS prepared figures. All authors interpreted results of the experiments and drafted, edited, and revised the manuscript, with JS and SM having the main contribution and approving the final version of the manuscript.

## Conflict of Interest Statement

The authors declare that the research was conducted in the absence of any commercial or financial relationships that could be construed as a potential conflict of interest.

## Footnotes

1 http://bioinfo.ut.ee/primer3-0.4.0/primer3/
