# Supplementary material for: Feeding Stimulation Ability and Central Effects of Intraperitoneal Treatment of L-Leucine, L-Valine, and L-Proline on Amino Acid Sensing Systems in Rainbow Trout: Implication in Food Intake Control
Source: Front Physiol. 2018 Aug 28;9:1209. doi: 10.3389/fphys.2018.01209 (PMC6121200; doi:10.3389/fphys.2018.01209)
Supplement: Supplementary file 1 [file Table_1.DOCX]

**TABLE 1** Nucleotide sequences of the PCR primers used to evaluate mRNA abundance by RT-PCR (qPCR).

|  | Forward primer | Reverse primer | Annealing temperature (°C) | Data base | Accession Number |
| --- | --- | --- | --- | --- | --- |
| β-actin | GATGGGCCAGAAAGACAGCTA | TCGTCCCAGTTGGTGACGAT | 59 | GenBank | NM_ 001124235.1 |
| AgRP | ACCAGCAGTCCTGTCTGGGTAA | AGTAGCAGATGGAGCCGAACA | 60 | GenBank | CR376289 |
| AMPKα1 | ATCTTCTTCACGCCCCAGTA | GGGAGCTCATCTTTGAACCA | 60 | GenBank | HQ40367 |
| BCKDE2 | CCCAAGATGAAGCCCACACC | CCAGGGAAGCAGCCTTGATG | 59 | GenBank | AB050595 |
| BCKDK | TGGACGACCACAAGGACGTG | AGACGGGAGGTGAGGGTGGT | 59 | Sigenae | BX076477.s.om.10. |
| CART | ACCATGGAGAGCTCCAG | GCGCACTGCTCTCCAA | 60 | GenBank | NM_001124627 |
| EF1α | TCCTCTTGGTCGTTTCGCTG | ACCCGAGGGACATCCTGTG | 59 | GenBank | AF498320 |
| eIF2α | TAGGATCGGACGCAATGAGTG | TCAGCCACGTGTCGAAGGAT | 59 | GenBank | AF338347.1 |
| GLS1 | CTGCAGTCTGTGTTCAGGGTAGA | CATCTGTCTGGAATTGTTAAGTCCATA | 58 | GenBank | AF390021 |
| GLS2 | GGCAGTGTCTTTAAATGGCAACA | ACGCTACAATTGGCAAGACTGA | 60 | GenBank | AF390022 |
| mTOR | ATGGTTCGATCACTGGTCATCA | TCCACTCTTGCCACAGAGAC | 60 | GenBank | EU179853 |
| NPY | CTCGTCTGGACCTTTATATGC | GTTCATCATATCTGGACTGTG | 58 | GenBank | NM_001124266 |
| POMC-A1 | CTCGCTGTCAAGACCTCAACTCT | GAGTTGGGTTGGAGATGGACCTC | 60 | Tigr | TC86162 |
| SESN2 | CTGCAGCTCGTCACCAGTGTT | GCAGCGTATTTTGGGGTGTGT | 59 | GenBank | XM_021572426.1 |
| SNAT2 | AAGGAGGGTCGCTGGTCTAT | GCATACCACTCTCCGTTGGT | 60 | GenBank | XM_020501247 |
| T1R1 | GAGTGGAATCAAGGCGATTGG | CTGGATGGCGTTTGTTCACAG | 59 | GenBank | XM_021614421.1 |
| T1R2 | GATGAGTGGGCCAGGAATGG | CCTCCCACCGGCTGACTTTA | 60 | Sigenae | FYV3OTN01AKLI0.s.om.10 |
| T1R3 | GCCCTGTGGAGCCCATCTTA | CCACACAGTAGGTCAGGGTGGA | 60 | Sigenae | GAY7CUQ01EHKNI.s.om.10 |
| AgRP, Agouti-related protein; AMPKα1, AMP-activated protein kinase α1; BCKDE2, branched chain α-keto acid dehydrogenase E2 subunit; BCKDK, branched chain α-keto acid dehydrogenase kinase; CART, cocaine- and amphetamine-related transcript; EF1α, elongation factor 1α; eIF2α, eukaryotic initiation factor 2α; GLS1, glutamine synthetase 1; GLS2, glutamine synthetase 2; mTOR, mechanistic target of rapamycin; NPY, neuropeptide Y; POMC-A1, pro-opio melanocortin A1; SESN2, Sestrin 2; SNAT2, system A amino acid transporter 2; T1R1, type 1 taste receptor subunit 1; T1R2, type 1 taste receptor subunit 2; T1R3, type 1 taste receptor subunit 3 | | | | | |
